# Extracellular DNA and Deoxyribonuclease Activity as Potential Biomarkers of Inflammation in Multiple Sclerosis

**DOI:** 10.1007/s12035-025-04907-4

**Published:** 2025-04-08

**Authors:** Peter Marček, Pavol Kadlic, Louise-Mária Adamová, Ľubomíra Tóthova, Michal Pastorek, Alexandra Gaál Kovalčíkova, Peter Valkovič, Michal Minár, Darina Slezáková

**Affiliations:** 1 Second Department of Neurology, Faculty of Medicine, Comenius University in Bratislava, Bratislava, Slovakia; 2 Institute of Molecular Biomedicine, Faculty of Medicine, Comenius University in Bratislava, Bratislava, Slovakia; 3 Centre of Experimental Medicine, Institute of Normal and Pathological Physiology, Slovak Academy of Sciences, Bratislava, Slovakia; 4 Department of Pediatrics, National Institute of Children’s Diseases and Faculty of Medicine, Comenius University in Bratislava, Bratislava, Slovakia

**Keywords:** Multiple sclerosis, Extracellular DNA, Deoxyribonuclease, Inflammation

## Abstract

Neuroinflammation plays a critical role in the pathophysiology of multiple sclerosis (MS), involving complex interactions between reactive oxygen species (ROS), cytokines, chemokines, and immune cells. Among these, neutrophils contribute to sustained inflammation through degranulation, ROS production, and the release of neutrophil extracellular traps (NETs). Extracellular DNA (ecDNA), a key component of NETs, may act as an autoantigen, promoting chronic inflammation and tissue damage. Additionally, impaired NETs and ecDNA degradation by deoxyribonucleases (DNases) may contribute to persistence of inflammation. The aim of the present study was to determine the levels of ecDNA and DNase activity in both blood plasma and cerebrospinal fluid (CSF) in newly diagnosed, treatment-naïve adult patients with relapsing–remitting MS and whether it correlates with disease severity and inflammatory activity in MS. Fifty-one treatment-naïve relapsing–remitting MS patients without disease-modifying therapy and 16 healthy controls (HC) were included in our study. Blood and CSF samples were analyzed for ecDNA, mitochondrial DNA (mtDNA) levels, and DNase activity. Correlations with inflammatory cytokines, oxidative stress, MRI lesion burden, and the expanded disability status scale (EDSS) were analyzed. MS patients exhibited significantly elevated ecDNA levels and reduced DNase activity in blood plasma compared to HC. EcDNA levels positively correlated with inflammatory cytokines, oxidative stress, and disease severity (EDSS). Furthermore, ecDNA and mtDNA levels in CSF positively correlated with inflammatory gadolinium-enhancing MRI lesions. Interestingly, no DNase activity was detected in CSF in both MS patients and HC. Our findings demonstrate that MS patients exhibit significantly elevated ecDNA levels and reduced DNase activity in blood plasma, which correlate with inflammatory cytokines, oxidative stress, and disease severity (EDSS). Additionally, increased ecDNA and mtDNA levels in CSF are associated with higher inflammatory activity, as reflected by gadolinium-enhancing MRI lesions. Considering the pro-inflammatory nature of ecDNA in perpetuating sterile inflammation, these results suggest a potential role of circulating nucleic acids in MS pathogenesis. Furthermore, impaired DNase activity may contribute to the persistence of ecDNA, potentially sustaining pro-inflammatory state in MS. Nevertheless, it remains unclear whether elevated ecDNA actively contributes to neuroinflammation or simply reflects ongoing immune activation. Further research is needed to elucidate the mechanisms underlying ecDNA release and degradation and its implications in MS progression.

**Supplementary Information:**

The online version contains supplementary material available at 10.1007/s12035-025-04907-4.

## Introduction

Multiple sclerosis (MS) is an autoimmune disease of the central nervous system (CNS) and represents the leading cause of the non-traumatic disability in people of productive age, with approximately 2.8 million individuals affected worldwide [1]. The pathogenesis of MS involves a complex interplay of neuroinflammatory and neurodegenerative processes that can operate independently, even during the preclinical phase, contributing to the heterogeneity in clinical manifestations and disease progression [2].

Neuroinflammation is a key driver of MS pathophysiology, characterized by complex interactions among reactive oxygen species (ROS), cytokines, chemokines, and immune cells, which contribute to persistent immune activation and CNS damage. Neutrophils play a particularly important role in this inflammatory cascade by degranulation, ROS production, and the release of neutrophil extracellular traps (NETs) [3]. Although overactivation of inflammation and immune dysregulation is known as the underlying factor in MS pathogenesis, the precise mechanisms remain incompletely understood.

Extracellular DNA (ecDNA) has emerged as an important mediator in inflammatory cascades, primarily released during apoptosis, necrosis, or by NETs formation by activated neutrophils [4–6]. This is supported by the fact, that ecDNA acts as a potent damage-associated molecular pattern (DAMP) that can engage pattern recognition receptors (PRRs), such as Toll-like receptor 9 (TLR- 9), to activate pro-inflammatory signaling pathways [7]. Activation of TLR- 9 by ecDNA drives the production of pro-inflammatory cytokines, which could perpetuate the inflammatory response and contribute to tissue damage [8]. Under physiological conditions, circulating ecDNA is efficiently cleared by deoxyribonucleases (DNases), thereby maintaining immune homeostasis [9].

The role of ecDNA in inflammatory diseases has been documented in previous studies. Elevated ecDNA levels have been observed in chemically induced colitis, correlating with neutrophil activation and intestinal inflammation [10]. Moreover, mitochondrial DNA (mtDNA) released during NETs formation by activated neutrophils has been shown to promote a pro-inflammatory state, contributing to disease progression [11]. Additionally, inadequate degradation of DNA-enriched NETs due to impaired DNase activity has been associated with the pathogenesis of lupus nephritis [12]. In fact, impaired DNase activity has been observed in several autoimmune diseases [13–15].

Neutrophils have been increasingly recognized as an important component in immune dysregulation in MS. It was recently described that patients with MS have an increased number of NETs in the serum when compared to healthy controls [16]. Additionally, neutrophils have been detected in the cerebrospinal fluid (CSF) of MS patients during the onset of disease and during the initiation phase of relapses, indicating their active involvement in the disease [17]. Since neutrophils are considered to be a major source of ecDNA in autoimmune disorders, their accumulation may play a role in perpetuating inflammation in MS. On the other hand, elevated ecDNA levels may just reflect ongoing neuroinflammatory and neurodegenerative processes as a consequence of immune activation. In addition, impaired DNase activity may contribute to the persistence of ecDNA, potentially sustaining a pro-inflammatory state. Although elevated ecDNA levels and impaired DNase activity have been observed in other autoimmune diseases, their association with disease activity in MS remains unclear.

To address this gap, the present study investigates ecDNA levels and DNase activity in both blood plasma and CSF of newly diagnosed, treatment-naïve patients with relapsing–remitting MS. Recognizing the potential of ecDNA to activate pro-inflammatory signaling pathways and possibly influence disease activity in MS, this study explores the relationship between ecDNA levels, inflammatory cytokines, oxidative stress markers, and their associations with MRI inflammatory lesions and disease severity as measured by the expanded disability status scale (EDSS).

## Patients and Methods

### Patients

In our study, we included consecutive adult patients from the Second Department of Neurology, Derer’s University Hospital, Bratislava, Slovakia, who were recently diagnosed with relapsing–remitting MS according to revised 2017 McDonald diagnostic criteria, treatment-naive without disease-modifying therapy (DMT) and without prior corticosteroid therapy and willing to participate. Patient who did not fulfill inclusion criteria, obese patients, and those with other comorbidities were excluded. Healthy control group consisted of age- and gender-matched individuals without neurological disorder or relevant comorbidity willing to participate.

### Methods

Informed consent was obtained in accordance with the Declaration of Helsinki and the local Ethics Committee (23/2021, Derer’s University Hospital, Bratislava, Slovakia) approved the study protocol. Relevant demographic and clinical data (age, gender, MS symptoms duration, EDSS) were collected. The venous peripheral blood samples were obtained in each MS patient in outpatient setting before the methylprednisolone treatment and the initiation of DMT. The blood samples were obtained also from healthy controls. The CSF samples were obtained before the methylprednisolone treatment during hospital admission as part of the diagnostic process in the part of the patients with MS. Majority of the MS patients underwent baseline MRI of the brain at the time of blood sampling. CSF samples were not obtained from healthy subjects, but respective parameters were compared with CSF from healthy subjects in sample biobank of the Second Department of Neurology, Derer’s University Hospital, Bratislava, Slovakia.

### Cytokine Analysis

The concentrations of cytokines (IL- 1β, IFN-α2, IFN-γ, TNF-α, MCP- 1, IL- 6, IL- 8, IL- 10, IL- 12p70, IL- 17 A, IL- 18, IL- 23, and IL- 33) were determined using the LEGENDplex™ Human Inflammation Panel (13-plex) in V-bottom plates (Biolegend, San Diego, CA, USA) and measured on a DxFlex cytometer (Beckman Coulter, Brea, CA, USA) according to the manufacturer’s instructions, as was previously published [18].

### ecDNA Isolation and Quantification

The venous blood and CSF samples were immediately centrifuged (1600 × *g* for 10 min at 4 °C), and supernatants were kept frozen until further analysis at − 80 °C. Prior to ecDNA isolation, samples were centrifuged again (16 000 × *g* for 10 min at 4 °C). DNA was isolated from 200 µl of EDTA plasma and CSF samples using a commercial kit (QIAamp DNA Mini Kit, Qiagen, Hilden, Germany) according to the manufacturer’s protocol and eluted into 50 µl of ultrapure water. DNA samples were stored at − 20 °C for further analysis. The concentration of total ecDNA was quantified using a fluorometric method according to the manufacturer’s protocol (Qubit Fluorometer and Qubit dsDNA HS Assay Kit (Invitrogen, Carlsbad, CA, USA). mtDNA in isolates were quantified using real-time polymerase chain reaction (real-time PCR) on the on the QTower3 (Analytik Jena GmbH, Jena, Germany) using the SsoAdvanced Universal SYBR Green Supermix (Bio-Rad, Hercules, CA, USA). For isolates from clinical samples, human beta-globin gene (F: 5′- GCT TCT GAC ACA ACT GTG TTC- 3′, R: 5′- CAC CAA CTT CAT CCA CGT TCA- 3′) and primer targeting D-loop (R: 5′- GAG GGG TGG CTT TGG AGT- 3′) amplification were used to quantify mtDNA. The whole protocol was previously published [19].

### DNase Activity Measurement

Single radial enzyme-diffusion (SRED) with GoodView Nucleic Acid Stain (SBS Genetech, Beijing, China) was used for the measurement of DNase activity in lithium-heparin plasma. One microliter of each sample was analyzed in 1% agarose gel (2 mM MgCl_2_, 2 mM CaCl_2_, 20 mM Tris–HCl pH 7.5, 0.035 mg/mL DNA isolated from porcine livers). The calibration curve was made of DNase I standard of known DNase activity, with serial twofold dilutions in the RDD buffer presented in the set (RNase-free DNase set, Qiagen, Hilden, Germany). After overnight incubation of gel (16–20 h, in the dark) at 37 °C, the gel was visualized in iBOX (Vision works LP Analysis Software, UVP, Upland, CA, USA). The circle diameters were measured using ImageJ software (NIH, Bethesda, Maryland, MD, USA). DNase activity calculation was based on comparison to the calibration curve. The activity was expressed in Kunitz units (K.u.) per mL of sample, as evaluated in other studies [20, 21].

### Assessment of Markers of Oxidative Stress and Antioxidant Status

As a marker of carbonyl stress, advanced glycation end products (AGEs) were measured. Briefly, 20 µl of samples and standards (AGEs-BSA) were mixed with 180 µl of PBS in a dark microtiter plate. AGEs were determined fluorometrically at _ex_. = 370 nm and _em_. = 440 nm as described previously [22]. For fructosamines measurement, 20 µl of samples and standards (1-deoxy-morpholino-d-fructose) were mixed with 100 µl of 0.25 mM/l nitro blue tetrazolium. Plates were incubated at 37 °C for 15 min, and absorbance was measured at 530 nm. As a marker of oxidative damage of proteins, advanced oxidation protein products (AOPP) were assessed as described previously [23]. Two hundred µl of samples and standards (chloramine T mixed with 10 µl of potassium iodide) were mixed with 20 μl of glacial acetic acid. Plates were shaken for 2 min, and absorbance was measured at 340 nm. As a marker of lipid peroxidation thiobarbituric acid reactive substances (TBARS) were evaluated, 20 µl of samples and standards (1,1,3,3-tetraethoxypropane) were mixed with 30 µl of distilled water, 20 µl of freshly prepared 0.67% thiobarbituric acid, and 20 µl of glacial acetic acid in PCR plate. Plates were incubated at 95 °C for 45 min. Afterwards, 100 µl of n-butanol were added and plates were centrifuged at 2000 g on 4 °C for 10 min. Seventy µl of upper phase were transferred into the microtiter plate and TBARS were determined fluorometrically at _ex._ = 515 nm and _em._ = 535 nm [24]. As markers of antioxidant status, total antioxidant capacity (TAC) and ferric reducing ability of plasma (FRAP) was assessed [25, 26]. For TAC measurement, 200 µl of acetate buffer (pH = 5.8) were mixed with 20 µl of samples and standards (trolox). Absorbance was measured at 660 nm and served as a blank. Afterwards, 2,2´azino-bis (ethylbenzthiazoline- 6-sulfonic acid) reagent was added, and absorbance was measured again. The initial absorbance was subtracted from the second measurement. For FRAP measurement, 200 µl of warmed FRAP reagent (acetate buffer (pH = 3.6), tripyridyl-s-triazine, FeCl_3_ ∗ 6H_2_O, water) were pipetted into microtiter plate. Absorbance was measured at 530 nm. Afterward, 20 µl of samples and standards (FeSO_4_ * 7H_2_O) were added, and absorbance was measured again. Concentration of plasma proteins was measured using commercial assay (BCA protein assay Macro Kit, Serva, Heidelberg, Germany). Ten microliters of samples and standards (bovine serum albumin) were mixed with 200 µl of fresh prepared working solution (bicinchoninic acid, copper sulfate, 49:1 ratio). The mixture was incubated at 37 °C for 30 min, and absorbance was measured at 562 nm, as evaluated elsewhere [27].

### MRI

Every MS patient underwent MRI (Philips Ingenia Elition 3,0 T X) of the brain with gadolinium contrast agent prior to CSF and blood sampling as part of the diagnostic process. All MRI examinations were performed using the same hardware and software throughout the whole study duration. Data was assessed by neuroradiologists trained in MS. Visual evaluation of the brain focused on detecting lesions (Flair/T2 hyperintensities, T1 hypointensities) and distribution in space, the detection of new Flair/T2 lesions, and contrast-enhancing T1 hyperintensities to evaluate disease activity—distribution in time, according to the 2017 McDonald criteria. Description of atrophic changes was assessed using visual rating scales. Simultaneously, MRI scans (3D Flair, 3D T1) were analyzed by Icobrain MS software (Icometrix, Leuven, Belgium). Icobrain MS software can detect, quantify, and track the evolution of MS lesions (Flair, T1) and distribution in space and time. The software provides metrics that can assess the volume of the whole brain and grey matter, tracking annualized brain volume changes to evaluate disease progression. Additionally, the software compares brain volume and volume changes to age—and sex—having matched a normative reference population. The calculated volumes can be used to interpret the subjects’ measurements considering a normative population.

### Statistical Analysis

The data were analyzed by JASP Team (2021) JASP (Version 0.16) software. Data normality was checked by the Shapiro–Wilk test. Descriptive statistics were used to evaluate the demographic and clinical data. Quantitative data were described as median values with their corresponding interquartile range. Categorical parametric data were presented as percentages. To compare quantitative data between two groups, we used Mann–Whitney *U* test; the chi square test was used for categorical variables. Correlation analysis of the data was conducted with the Spearman correlation test. If not stated otherwise, a limit level of significance was considered when *p* values were less than 0.05. Using the Bonferroni approach to control type I errors, a *p* value ≤ 0.002 was required for statistical significance.

## Results

We have collected and analyzed complete data from 51 patients (39 F/12 M) with relapsing–remitting MS and 16 age- and gender-matched healthy controls (13 F/3 M). The median of age was 31.5 (IQR 15.5) in HC and 34.0 (IQR 13.5) in MS group (*p* = ns). There were 76.5% of females in MS group (*n* = 39), and 81.3% females (*n* = 13) in the control group. In MS group, median of disease duration was 9.0 (IQR 28.2) months, median EDSS was 3.0 (IQR 2.0). Demographic data of patients with MS and healthy controls are shown in Table 1.

**Table 1:** Demographic data of patients with MS and healthy controls

	RRMS	HC
Gender	39 F/12 M	13 F/3 M
	**Mean ± STDEV**	
Age	34.137 ± 9.531	36.188 ± 10.653
MS duration from 1 st symptom till dg. [months]	32.369 ± 53.180	
EDSS	2.878 ± 1.210	
T2 supratentorial lesion load	21.565 ± 14.409	
T2 infratentorial lesion load	2.478 ± 3.356	
T2 total lesion load	24.043 ± 16.748	
Flair lesion volume [ml]	4.650 ± 4.680	
T1 lesion volume [ml]	2.892 ± 3.349	
Oligoclonal bands in CSF	Present in all patients	

*RRMS* Relapsing–remitting multiple sclerosis, *HC* healthy controls, *EDSS* expanded disability status scale, *IQR* interquartile range, *T1* T1 weighted image magnetic resonance, *T2* T2 weighted image magnetic resonance, *CSF* cerebrospinal fluid

### Blood Samples

**Fig. 1:**
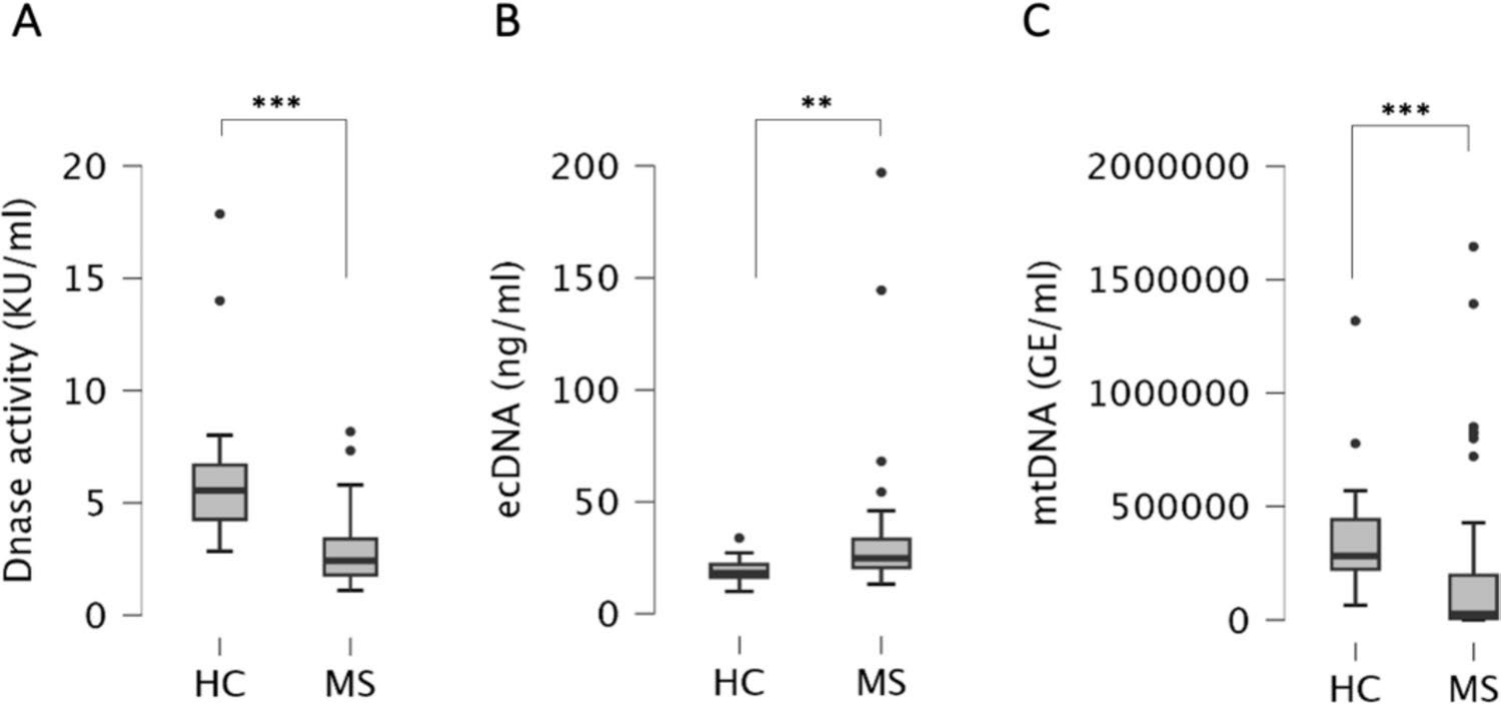
Comparison of blood DNase activity (**A**), ecDNA levels (**B**), and mtDNA levels (**C**) between healthy controls (HC) and patients with multiple sclerosis (MS). **p < 0.01, ***p < 0.001

Compared to HC group, patients with MS had significantly lower blood DNase activity (5.56 KU/ml IQR = 2.42 vs. 2.42 KU/ml IQR = 1.61; *p* < 0.001; Fig. 1A); significantly higher ecDNA levels (18.2 ng/ml IQR = 5.7 vs. 24.8 ng/ml IQR = 12.7; *p* < 0.01; Fig. 1B) and significantly lower mtDNA levels (27,746.1 GE/ml IQR = 190,241.2 vs. 283,246.3 GE/ml IQR = 218,731.6; *p* < 0.001; Fig. 1C).

Patients with MS had significantly higher markers of inflammation (IL- 1β, IL- 8, IL- 10, IL- 17 A, IL- 18, and MCP- 1), lipid peroxidation (TBARS), and total antioxidant capacity (TAC), compared to HC. All data are summarized in Table 2.

**Table 2:** Difference between inflammatory cytokines and oxidative stress in MS and HC in blood plasma (after Bonferroni correction, the level of significance was *p* < 0.002)

Parameter	HC		MS		
	Median	IQR	Median	IQR	p
IL- 1β [pg/ml]	1.150	1.692	6.980	7.235	** < 0.001**
IFN-α2 [pg/ml]	1.115	2.150	2.450	3.545	< 0.05
IFN-γ [pg/ml]	3.190	5.455	4.310	7.505	ns
TNF-α [pg/ml]	1.805	2.775	4.940	9.915	< 0.05
MCP- 1 [pg/ml]	39.645	22.795	130.070	152.110	** < 0.001**
IL- 6 [pg/ml]	2.845	5.488	8.470	7.565	< 0.01
IL- 8 [pg/ml]	0.290	0.267	7.080	24.315	** < 0.001**
IL- 10 [pg/ml]	1.215	2.737	5.810	10.005	** < 0.002**
IL- 12p70 [pg/ml]	1.905	3.148	4.990	5.275	< 0.05
IL- 17 A [pg/ml]	0.180	0.448	0.880	1.095	** < 0.001**
IL- 18 [pg/ml]	79.580	47.575	146.360	155.185	** < 0.001**
IL- 23 [pg/ml]	1.840	2.917	2.560	6.015	ns
IL- 33 [pg/ml]	17.990	43.312	66.920	85.005	< 0.01
AGEs [g/l]	1.421	0.115	1.599	0.328	< 0.05
Fructosamine [mmol/l]	1.580	0.370	1.690	0.575	ns
AOPP [µmol/l]	58.090	38.400	95.950	65.300	ns
TBARS [µmol/l]	1.155	1.083	32.100	70.415	** < 0.001**
FRAP [µmol/l]	952.375	145.460	912.647	205.498	ns
TAC [µmol/l]	1655.500	260.250	1979.000	415.000	** < 0.001**

*MS* Multiple sclerosis, *HC* healthy controls, *IQR* interquartile range, *ecDNA* extracellular DNA, *mtDNA* mitochondrial DNA, *DNase* deoxyribonuclease, *IL* interleukin, *IFN-γ* interferon, *TNF-α* tumor necrosis factor AGEs—advanced glycation end products, *AOPP* advanced oxidation protein products, *TBARS* thiobarbituric acid reactive substances, *FRAP* ferric reducing ability of plasma, *TAC* total antioxidant capacity

There was no gender-related difference in mtDNA, ecDNA, or DNase activity. In MS group we also did not observe correlation among DNase activity and level of ecDNA and mtDNA as well as no correlation with MS symptoms duration. However, DNase activity negatively correlated with age (*r*_*s*_ = − 0.3498) so all the following analyses of DNase activity in blood were controlled for age (Table 3). Taking those four parameters into account, Bonferroni adjusted *p* value less than 0.0125 (0.05/4) should be considered significant. Nevertheless, DNase activity inversely correlated with age only in MS patients (*p* < 0.01), not in HC (*p* = 0.841).

**Table 3:** Correlation among ecDNA, mtDNA and DNase activity in blood plasma in MS with age and MS symptoms duration. Data reported as Spearman’s rho (*r*_*s*_). *p < 0.05

	DNase activity [KU/ml]	ecDNA [ng/ml]	mtDNA [GE/ml]
DNase activity [KU/ml]	—		
ecDNA [ng/ml]	− 0.0852	—	
mtDNA [GE/ml]	0.0443	0.0620	—
Age [years]	− 0.3498*	0.0198	0.1772
MS symptoms duration [months]	− 0.1743	− 0.0229	0.0424

*MS* Multiple sclerosis, *ecDNA* extracellular DNA, *mtDNA* mitochondrial DNA, *DNase* deoxyribonuclease, *EDSS* expanded disability status scale

**Fig. 2:**
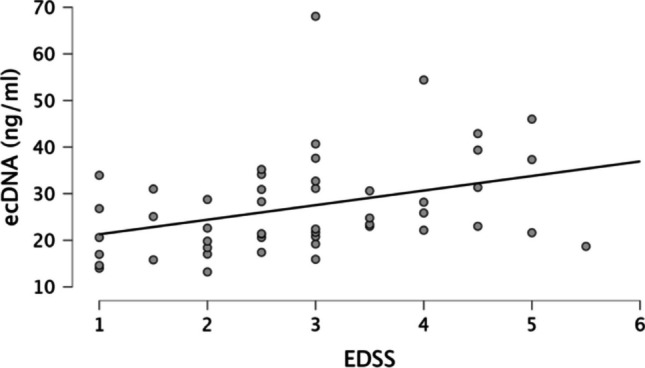
Correlation of increased ecDNA levels in blood plasma with higher disease severity measured by EDSS in patients with MS

Correlation data are shown in Table 4. Shortly, in MS group, DNase activity negatively correlated with IL- 1b (*r*_*s*_ = − 0.4077, *p* < 0.01) and IL- 18 (*r*_*s*_ = − 0.3656, *p* < 0.01). Levels of ecDNA positively correlated with IL- 8 (*r*_*s*_ = 0.2880, *p* < 0.05), MCP- 1 (*r*_*s*_ = 0.3359, *p* < 0.05), IL- 18 (*r*_*s*_ = 0.3017, *p* < 0.05), TBARS (*r*_*s*_ = 0.3418, *p* < 0.05, TAC (*r*_*s*_ = 0.4009, *p* < 0.01). Scatter plots for certain inflammatory cytokines are shown in Supplementary Figs. 1–3. Levels of mtDNA negatively correlated with MCP- 1 (*r*_*s*_ = − 0.5788, *p* < 0.001), IL- 8 (*r*_*s*_ = − 0.6627, *p* < 0.001), IL- 10 (*r*_*s*_ = − 0.3821, *p* < 0.01), IL- 18 (*r*_*s*_ = − 0.420, *p* < 0.01), TBARS (*r*_*s*_ = − 0.6142, *p* < 0.001), and TAC (*r*_*s*_ = − 0.2892, *p* < 0.05). Interestingly, ecDNA positively correlated with EDSS (*r*_*s*_ = 0.4647, *p* < 0.001; Fig. 2). There was no other correlation between ecDNA, mtDNA and DNase activity and MRI findings or EDSS. All other correlations are presented in Supplementary Table 1, along with data on healthy subjects that are presented in Supplementary Table 2 and 3.

**Table 4:** Correlation between ecDNA, mtDNA and DNase activity in blood plasma in multiple sclerosis patients and inflammatory cytokines, and oxidative stress parameters. Data presented as Spearman’s rho (*r*_*s*_). *p < 0.05, **p < 0.01, and ***p < 0.001

	Dnase activity [KU/ml]	ecDNA [ng/ml]	mtDNA [GE/ml]
IL- 1β [pg/ml]	− 0.4077**	0.06095	− 0.2345
MCP- 1 [pg/ml]	− 0.2447	0.3359*	− 0.5788***
IL- 8 [pg/ml]	− 0.2030	0.3363*	− 0.6627***
IL- 10 [pg/ml]	− 0.1780	0.09104	− 0.3821**
IL- 17 A [pg/ml]	− 0.1223	− 1.810e − 4	− 0.2527
IL- 18 [pg/ml]	− 0.3656**	0.3017*	− 0.4201**
TBARS [µmol/l]	− 0.1706	0.3418*	− 0.6142***
TAC [µmol/l]	− 0.2134	0.4009**	− 0.2892*

*ecDNA* Extracellular DNA, *mtDNA* mitochondrial DNA, *DNase* deoxyribonuclease, *TBARS* thiobarbituric acid reactive substances, *TAC* total antioxidant capacity

### CSF Samples

We obtained 30 CSF samples from our cohort of MS patients. The control group consisted of 15 healthy adults without inflammatory or neurodegenerative disorder whose CSF samples were available in our CSF biobank. There were no blood samples from the control group.

There was no significant difference in age between HC and MS group. The median age was 32.0 (IQR = 6.5) and 31.5 (IQR = 11.5) for HC and MS group, respectively. There were 60.7% (*n* = 10) females in the control group and 80% (*n* = 24) of females in MS group.

No DNase activity was detected either in MS patients or control group in CSF. In case of ecDNA and mtDNA (Supplementary Fig. 4 A and B), no significant difference was present in CSF between MS and control samples. Several of the inflammatory cytokines in CSF (IL- 23, IFN- α2, TNF- α, IL- 10, and IL- 17 A) were higher in patients with MS compared to HC, however, did not reach statistical significance. Data are summarized in Supplementary Table 4.

**Fig. 3:**
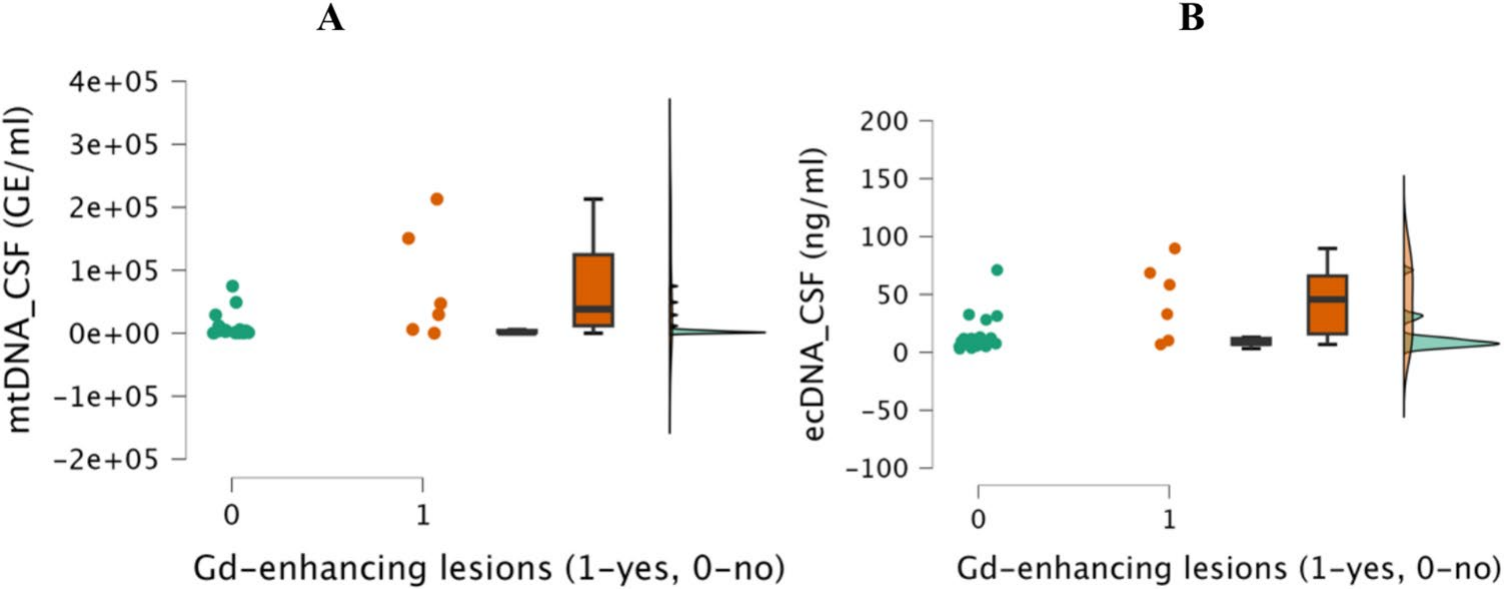
Comparison of CSF mtDNA levels (**A**) and CSF ecDNA levels (**B**) in patients with MS with and without inflammatory gadolinium-enhancing MRI lesions

Interestingly, MS patients with inflammatory gadolinium-enhancing MRI lesions had significantly higher mtDNA and ecDNA levels in CSF—38,260.01 (IQR 112818.96) vs. 1520.15 GE/ml (IQR 3370.11), *p* = 0.041; and 45.66 (IQR 50.08) vs. 8.14 ng/ml (IQR 5.16), *p* = 0.031—Fig. 3. Furthermore, patients with high MRI lesion load with more than nine T2 MRI lesions had higher mtDNA levels in CSF—4326.11 (IQR 32643.85) vs. 1103.7 GE/ml (IQR 1326.54), *p* = 0.043—Supplementary Fig. 5.

## Discussion

Neuroinflammation is an important contributor to the pathology of MS and includes several different factors—reactive oxygen species (ROS), cytokines and chemokines produced via endothelial cells, microglia, astrocytes, or peripheral immune cells, which in turn contribute to chronic inflammation, promoting neuronal injury and cell death. Neutrophils are among the first migrating immune cells from the periphery to the affected regions in the CNS. By releasing cytokines, metalloproteinases, ROS and NETs, as well as attracting additional immune cells, neutrophils contribute to the persistence and progression of the inflammatory response [28]. Degranulation, phagocytosis, and ROS production are the well-known mechanisms. However, neutrophils can release fibrous web-like structures composed of DNA, antimicrobial granules and proteins. These complexes should help to entrap and neutralize pathogens. Nevertheless, components of NETs released into extracellular space can be recognized as auto-antigens, potentially triggering an inflammatory response and disrupting self-tolerance [29]. Additionally, an imbalance between NETs formation and degradation may prolong immune system exposure to these auto-antigens, which further exacerbates the NETs-induced tissue damage [30].

### Blood Plasma

Our study demonstrated that total extracellular DNA (ecDNA) levels in the blood plasma of multiple sclerosis (MS) patients were almost twice as high as those in healthy controls (HC). Additionally, ecDNA levels positively correlated with inflammatory cytokines and oxidative stress, both of which were significantly increased in the MS group. Furthermore, higher ecDNA levels were linked with higher disease severity, as measured by expanded disability status scale (EDSS). These findings highlight the potential role of ecDNA in MS-related inflammation and disease progression. Additionally, our findings revealed significantly reduced DNase activity in MS patients compared to HC, with DNase activity showing a negative correlation with both age and inflammatory cytokines. This suggests that impaired DNase function may contribute to persistent ecDNA accumulation, potentially sustaining the inflammatory response in MS.

Neutrophils, which are considered key players in autoimmune disorders, can be activated by ecDNA, contributing to the pathophysiology of MS. While excessive neutrophil activation may exacerbate tissue damage and chronic inflammation, it is important to determine whether elevated ecDNA actively drives this activation or simply reflects ongoing inflammatory response. Considering that ecDNA can activate pattern recognition receptors such as Toll-like receptor 9 (TLR- 9), triggering downstream immune responses, it is plausible that ecDNA serves as more than just a biomarker of inflammation and may actively contribute to disease progression. This is supported by the fact that impaired DNase activity observed in our MS group suggests its pro-inflammatory nature. These findings are consistent with other studies showing that exogenous DNase administration can counteract the pro-inflammatory effects of ecDNA and NETs. In the case of NETs, such an approach disrupts the trap structure, thereby reducing the pro-inflammatory effect. In recent years, DNase administration has been shown to be effective in animal models, for example in organ damage induced by an experimental model of sepsis [31]. On the other hand, the lack of a direct correlation between DNase activity and ecDNA levels in our study highlights the significance of alternative pathways for ecDNA degradation, such as macrophage-mediated degradation or as a consequence of DNase inhibitors [32, 33]. While circulating DNase I plays a key role in clearing ecDNA from the bloodstream, another crucial mechanism involves phagocytic processing. Once ecDNA is engulfed by phagocytes, it is transported to the lysosome, where DNase II serves as the primary enzyme responsible for its degradation [34]. As a result, these mechanisms may have played a role in the accumulation of ecDNA levels. Nevertheless, the negative correlation between DNase activity and inflammatory cytokines in our study suggests that reduced DNase activity in the MS group may have played a role in ecDNA accumulation, potentially driving inflammation. Further research is needed to investigate the contribution of alternative clearance pathways in ecDNA degradation in MS.

Analysis of the subcellular origin of ecDNA in blood plasma revealed significantly decreased mtDNA levels in MS patients compared to healthy controls (HC). Unlike nuclear DNA, mtDNA is highly susceptible to oxidative damage from ROS due to its close proximity to ROS within the mitochondrial matrix, lack of protective histones, and limited DNA repair capacity [35, 36]. Our findings support this susceptibility, as lower mtDNA levels in the blood correlated with increased oxidative stress, whereas higher antioxidative capacity was associated with elevated mtDNA levels in the MS cohort. These findings align partially with a previous study, which reported decreased mtDNA levels in the peripheral blood of relapsing–remitting MS patients receiving disease-modifying therapy [37]. However, given the limited evidence on mtDNA levels in blood in MS, the factors underlying this phenomenon remain unclear and warrant further investigation. However, given that MS is characterized as a neuroinflammatory disease, the observed discrepancy in our results between decreased mtDNA levels in blood and increased mtDNA in CSF in patients with high inflammatory activity on MRI, may reflect the localized nature of inflammation within the CNS.

### CSF

Previous studies have reported a significant elevation of mtDNA in CSF in relapsing–remitting MS and suggested its association with inflammatory activity [38]. In our MS cohort, ecDNA and mtDNA levels in CSF were higher than in healthy controls, although these differences did not reach statistical significance. However, higher CSF mtDNA levels were significantly associated with active inflammatory gadolinium-enhancing MRI lesions and increased lesion burden, reinforcing its potential link to disease activity.

Our results align with previous studies, demonstrating elevated mtDNA levels in CSF as a marker of disease activity in relapsing–remitting MS and their dynamic response to autologous hematopoietic stem cell transplantation [39]. Notably, increased cell-free mtDNA in CSF during early stages of MS has been suggested as an important link between brain and immune system, particularly due to its ability to stimulate the inflammatory process [40]. Over time, chronic inflammation in the CNS may contribute to mitochondrial depletion within neurons, leading to lower mtDNA levels in progressive MS, as previously reported [41]. Interestingly, DNase activity was undetectable in CSF in both MS patients and healthy controls, which may contribute to the persistence of immunostimulatory mtDNA within the CNS compartment. However, the evidence regarding DNase activity in CSF and its role to mitigate inflammatory process is very scarce and requires further research.

Our study has some limitations. Other forms of circulating mtDNA, such as those encapsulated in extracellular vesicles, are present in the circulation, but these were not covered in our protocol. Furthermore, our study protocol did not analyze the sequence and fragment length patterns of DNA, which would enable us to further specify the source of ecDNA in our samples. Since our study group included only consecutive treatment-naïve MS patients, we did not evaluate the serial changes of circulating nucleic acids and DNase activity over time. Specifically, observing changes in circulating nucleic acids levels after the initiation of disease-modifying therapy could shed some light on the association between ecDNA, mtDNA, and DNase activity with inflammatory activity in MS. Additionally, we did not compare a control sample of similar size to the study population due to the absence of available control CSF samples in our biobank. Furthermore, the concentration of ecDNA is influenced by several pre-analytical physiological variables, including gender, obesity, menstruation, exercise, diet, and stress. In addition, technical factors such as collection tube type, centrifugation speed, and storage conditions can also affect the measured concentrations (42). Although these variables complicate direct comparisons between studies and the determination of pathological thresholds, they do not undermine the hypothesized proinflammatory properties of ecDNA, particularly when appropriate controls are employed.

In conclusion, our results demonstrate that ecDNA levels are significantly elevated in the blood plasma in early stages of MS and are associated with inflammatory activity and disease severity, as measured by EDSS. Additionally, mtDNA levels in CSF correlated with inflammatory gadolinium-enhancing MRI lesions and higher lesion burden, suggesting a potential link to disease activity. However, it remains unclear, whether elevated ecDNA levels actively contribute to the inflammatory process or simply reflect ongoing inflammation. Further research is needed to clarify the role of circulating nucleic acids and DNase activity in inflammatory activity in MS, particularly in the context of disease-modifying therapy.

## Supplementary Information

Below is the link to the electronic supplementary material.

Supplementary file1 (DOCX 288 KB)

## Data Availability

Data is provided within the manuscript or supplementary information files.
