# Supplementary material for: Extracellular DNA and Deoxyribonuclease Activity as Potential Biomarkers of Inflammation in Multiple Sclerosis
Source: Mol Neurobiol. 2025 Apr 8;62(8):10310–9. doi: 10.1007/s12035-025-04907-4 (PMC12289829; doi:10.1007/s12035-025-04907-4)
Supplement: Supplementary file 1 — Supplementary file1 (DOCX 288 KB) [file 12035_2025_4907_MOESM1_ESM.docx]

Supplementary table 1. Correlation between ecDNA, mtDNA and DNase activity in blood plasma in MS and inflammatory cytokines, oxidative stress and MRI parameters.

| **Variable** |  | **Dnase activity (KU/ml)** | **ecDNA (ng/ml)** | **mtDNA (GE/ml)** |
| --- | --- | --- | --- | --- |
| IFN-α2 (pg/ml) | Spearman's rho | -0.06963 | -0.1097 | -0.2628 |
|  | p-value | 0.631 | 0.443 | 0.062 |
| IFN-γ (pg/ml) | Spearman's rho | -0.2531 | -0.1978 | 0.01523 |
|  | p-value | 0.076 | 0.164 | 0.916 |
| TNF-α (pg/ml) | Spearman's rho | -0.07343 | -0.04937 | -0.1339 |
|  | p-value | 0.612 | 0.731 | 0.349 |
| IL-6 (pg/ml) | Spearman's rho | -0.06657 | 0.01774 | -0.3945 |
|  | p-value | 0.646 | 0.902 | 0.004** |
| IL-12p70 (pg/ml) | Spearman's rho | -0.2004 | -0.08756 | -0.03208 |
|  | p-value | 0.163 | 0.541 | 0.823 |
| IL-23 (pg/ml) | Spearman's rho | -0.2279 | -0.09575 | 0.2118 |
|  | p-value | 0.111 | 0.504 | 0.136 |
| IL-33 (pg/ml) | Spearman's rho | -0.1221 | -0.01407 | -0.4482 |
|  | p-value | 0.398 | 0.922 | < .001** |
| AGEs g/l | Spearman's rho | -0.09562 | 0.3250 | -0.6333 |
|  | p-value | 0.509 | 0.020** | < .001** |
| fructosamine mmol/l | Spearman's rho | -0.02253 | 0.2402 | -0.2433 |
|  | p-value | 0.877 | 0.090 | 0.085 |
| AOPP µmol/l | Spearman's rho | 0.06514 | 0.3314 | -0.4321 |
|  | p-value | 0.653 | 0.018** | 0.002** |
| FRAP µmol/l | Spearman's rho | 0.1565 | 0.01720 | 0.4561 |
|  | p-value | 0.278 | 0.905 | < .001** |
| T2 supratentorial lesion load | Spearman's rho | -0.1333 | 0.1885 | -0.04020 |
|  | p-value | 0.554 | 0.389 | 0.855 |
| T2 infratentorial lesion load | Spearman's rho | -0.1569 | 0.2977 | 0.2539 |
|  | p-value | 0.486 | 0.168 | 0.242 |
| T2 | Spearman's rho | -0.1425 | 0.2143 | 0.01015 |
|  | p-value | 0.527 | 0.326 | 0.963 |
| Flair lesion volume | Spearman's rho | 0.04950 | -0.1304 | 0.02372 |
|  | p-value | 0.831 | 0.561 | 0.917 |
| T1 lesion volume | Spearman's rho | -0.09495 | -0.09430 | 0.01864 |
|  | p-value | 0.682 | 0.676 | 0.934 |
| EDSS | Spearman's rho | -0.01552 | 0.4647 | -0.06878 |
|  | p-value | 0.914 | < .001** | 0.632 |

Abbreviations: HC – healthy controls, ecDNA – extracellular DNA, mtDNA – mitochondrial DNA, DNase – deoxyribonuclease, IFN-γ – interferon, TNF-α – tumor necrosis factor AGEs - advanced glycation end products, AOPP - advanced oxidation protein products, TBARS - thiobarbituric acid reactive substances, FRAP - ferric reducing ability of plasma, TAC – total antioxidant capacity, BCA – bicinchoninic acid

Supplementary table 2. Correlation among ecDNA, mtDNA and DNase activity in blood plasma in HC

| **Variable** |  | **Dnase activity (KU/ml)** | **mtDNA (GE/ml)** | **ecDNA (ng/ml)** |
| --- | --- | --- | --- | --- |
| mtDNA (GE/ml) | Spearman's rho | 0.2147 | — |  |
|  | p-value | 0.423 | — |  |
| ecDNA (ng/ml) | Spearman's rho | 0.2353 | 0.2618 | — |
|  | p-value | 0.379 | 0.326 | — |
| Age | Spearman's rho | -0.05461 | 0.1358 | -0.1875 |
|  | p-value | 0.841 | 0.616 | 0.487 |

Abbreviations: mtDNA – mitochondrial DNA, ecDNA – extracellular DNA, DNase - deoxyribonuclease

Supplementary table 3. Correlation between ecDNA, mtDNA and DNase activity in blood plasma in HC and inflammatory cytokines and oxidative stress

| **Variable** |  | **Dnase activity (KU/ml)** | **ecDNA (ng/ml)** | **mtDNA (GE/ml)** |
| --- | --- | --- | --- | --- |
| IL-1β (pg/ml) | Spearman's rho | -0.4882 | -0.3912 | -0.2324 |
|  | p-value | 0.057 | 0.135 | 0.385 |
| IFN-α2 (pg/ml) | Spearman's rho | -0.3412 | 0.02059 | -0.01471 |
|  | p-value | 0.196 | 0.943 | 0.961 |
| IFN-γ (pg/ml) | Spearman's rho | -0.4765 | 0.07059 | 0.04118 |
|  | p-value | 0.064 | 0.797 | 0.882 |
| TNF-α (pg/ml) | Spearman's rho | -0.1441 | -0.2147 | 0.1853 |
|  | p-value | 0.594 | 0.423 | 0.491 |
| MCP-1 (pg/ml) | Spearman's rho | -0.2853 | -0.4765 | -0.1912 |
|  | p-value | 0.283 | 0.064 | 0.477 |
| IL-6 (pg/ml) | Spearman's rho | -0.3676 | 0.08529 | -0.005882 |
|  | p-value | 0.162 | 0.755 | 0.987 |
| IL-8 (pg/ml) | Spearman's rho | -0.3770 | -0.2312 | 0.1959 |
|  | p-value | 0.150 | 0.389 | 0.467 |
| IL-10 (pg/ml) | Spearman's rho | -0.4118 | -0.1118 | -0.1735 |
|  | p-value | 0.114 | 0.681 | 0.519 |
| IL-12p70 (pg/ml) | Spearman's rho | -0.3618 | 0.1118 | -0.02059 |
|  | p-value | 0.169 | 0.681 | 0.943 |
| IL-17A (pg/ml) | Spearman's rho | -0.4374 | -0.09278 | 0.2150 |
|  | p-value | 0.090 | 0.733 | 0.424 |
| IL-18 (pg/ml) | Spearman's rho | -0.4500 | -0.05000 | -0.2382 |
|  | p-value | 0.082 | 0.856 | 0.373 |
| IL-23 (pg/ml) | Spearman's rho | -0.6088 | -0.1471 | -0.06176 |
|  | p-value | 0.014** | 0.586 | 0.822 |
| IL-33 (pg/ml) | Spearman's rho | -0.4824 | 0.02647 | -0.07647 |
|  | p-value | 0.061 | 0.926 | 0.780 |
| AGEs g/l | Spearman's rho | 0.2035 | 0.06490 | 0.005900 |
|  | p-value | 0.450 | 0.811 | 0.983 |
| AGEs RFU | Spearman's rho | 0.2265 | 0.06471 | 0.02059 |
|  | p-value | 0.398 | 0.814 | 0.943 |
| fructosamine mmol/l | Spearman's rho | 0.3247 | -0.1033 | 0.4236 |
|  | p-value | 0.220 | 0.703 | 0.102 |
| AOPP µmol/l | Spearman's rho | 0.2870 | -0.09272 | 0.3974 |
|  | p-value | 0.281 | 0.733 | 0.128 |
| TBARS µmol/l | Spearman's rho | 0.1441 | -0.4676 | -0.3353 |
|  | p-value | 0.594 | 0.070 | 0.204 |
| FRAP µmol/l | Spearman's rho | -0.06471 | 0.1647 | -0.4676 |
|  | p-value | 0.814 | 0.541 | 0.070 |
| TAC µmol/l | Spearman's rho | -0.3118 | -0.3912 | -0.5941** |
|  | p-value | 0.239 | 0.135 | 0.017 |
| BCA g/l | Spearman's rho | -0.1735 | -0.2294 | -0.2412 |
|  | p-value | 0.519 | 0.391 | 0.367 |
| AGEs g/g proteins | Spearman's rho | 0.1274 | 0.1798 | 0.1244 |
|  | p-value | 0.638 | 0.505 | 0.646 |
| AGEs RFU/g proteins | Spearman's rho | 0.1794 | 0.1235 | 0.09706 |
|  | p-value | 0.505 | 0.648 | 0.721 |
| fructosamine mmol/g proteins | Spearman's rho | 0.1428 | -0.06770 | 0.5475** |
|  | p-value | 0.598 | 0.803 | 0.028 |
| AOPP µmol/g proteins | Spearman's rho | 0.3032 | -0.1148 | 0.4503 |
|  | p-value | 0.254 | 0.672 | 0.080 |
| TBARS µmol/g proteins | Spearman's rho | 0.06342 | -0.5327 | -0.1316 |
|  | p-value | 0.815 | 0.034** | 0.627 |
| FRAP µmol/g proteins | Spearman's rho | -0.1647 | 0.2735 | -0.4471 |
|  | p-value | 0.541 | 0.304 | 0.084 |
| TAC µmol/g proteins | Spearman's rho | -0.5000 | -0.2618 | -0.5088** |
|  | p-value | 0.051 | 0.326 | 0.046 |

Abbreviations: HC – healthy controls, ecDNA – extracellular DNA, mtDNA – mitochondrial DNA, DNase – deoxyribonuclease, IFN-γ – interferon, TNF-α – tumor necrosis factor AGEs - advanced glycation end products, AOPP - advanced oxidation protein products, TBARS - thiobarbituric acid reactive substances, FRAP - ferric reducing ability of plasma, TAC – total antioxidant capacity, BCA – bicinchoninic acid

Supplementary table 4. Difference between ecDNA, mtDNA, DNase activity, inflammatory cytokines and oxidative stress in MS and control group in CSF

|  | **Group** | **Median** | **IQR** | **Mean** | **Std. Deviation** | **p** |
| --- | --- | --- | --- | --- | --- | --- |
| mtDNA_CSF (GE/ml) | HC | 1332.574 | 6594.509 |  |  |  |
|  | MS | 3305.989 | 9238.613 |  |  | 0.576 |
| ecDNA_CSF (ng/ml) | HC | 7.620 | 1.995 |  |  |  |
|  | MS | 9.825 | 17.142 |  |  | 0.099 |
| IL-1β (pg/ml) | HC | 0.121 | 0.165 |  |  |  |
|  | MS | 0.113 | 0.169 |  |  | 0.736 |
| IFN-α2 (pg/ml) | HC | 0.135 | 0.019 |  |  |  |
|  | MS | 0.148 | 0.029 |  |  | 0.099 |
| IFN-γ (pg/ml) | HC |  |  | 0.777 | 0.097 | 0.936 |
|  | MS |  |  | 0.780 | 0.104 |  |
| TNF-α (pg/ml) | HC | 0.580 | 0.282 |  |  |  |
|  | MS | 0.752 | 0.261 |  |  | 0.064 |
| MCP-1 (pg/ml) | HC | 383.419 | 176.629 |  |  |  |
|  | MS | 206.095 | 214.210 |  |  | 0.002** |
| IL-6 (pg/ml) | HC | 1.533 | 2.721 |  |  |  |
|  | MS | 0.593 | 0.635 |  |  | 0.147 |
| IL-8 (pg/ml) | HC | 4.318 | 7.318 |  |  |  |
|  | MS | 5.394 | 8.566 |  |  | 0.782 |
| IL-10 (pg/ml) | HC | 0.087 | 0.058 |  |  |  |
|  | MS | 0.118 | 0.084 |  |  | 0.087 |
| IL-12p70 (pg/ml) | HC | 0.096 | 0.028 |  |  |  |
|  | MS | 0.108 | 0.053 |  |  | 0.206 |
| IL-17A (pg/ml) | HC | 0.007 | 0.005 |  |  |  |
|  | MS | 0.010 | 0.008 |  |  | 0.040 |
| IL-18 (pg/ml) | HC | 0.596 | 0.396 |  |  |  |
|  | MS | 0.398 | 0.296 |  |  | 0.453 |
| IL-23 (pg/ml) | HC | 0.287 | 0.163 |  |  |  |
|  | MS | 0.360 | 0.168 |  |  | 0.047 |
| IL-33 (pg/ml) | HC | 2.250 | 0.770 |  |  |  |
|  | MS | 2.510 | 0.731 |  |  | 0.358 |
| AGEs g/l | HC | 0.033 | 0.042 |  |  |  |
|  | MS | 0.029 | 0.022 |  |  | 0.523 |
| fructosamines mmol/l | HC | 0.000 | 0.041 |  |  |  |
|  | MS | 0.000 | 0.127 |  |  | 0.775 |
| AOPP µmol/l | HC | 0.000 | 0.000 |  |  |  |
|  | MS | 0.000 | 0.386 |  |  | 0.121 |
| TBARS µmol/l | HC |  |  | 0.023 | 0.016 | 0.698 |
|  | MS |  |  | 0.025 | 0.016 |  |
| FRAP µmol/l | HC | 116.870 | 135.095 |  |  |  |
|  | MS | 114.742 | 152.824 |  |  | 0.605 |
| TAC µmol/l | HC |  |  | 259.458 | 108.462 | 0.869 |
|  | MS |  |  | 264.489 | 88.603 |  |

Abbreviations: MS – multiple sclerosis, HC – healthy controls, SD – standard deviation, IQR – interquartile range, ecDNA – extracellular DNA, mtDNA – mitochondrial DNA, DNase – deoxyribonuclease, CSF – cerebrospinal fluid, IFN-γ – interferon, TNF-α – tumor necrosis factor AGEs - advanced glycation end products, AOPP - advanced oxidation protein products, TBARS - thiobarbituric acid reactive substances, FRAP - ferric reducing ability of plasma, TAC – total antioxidant capacity, BCA – bicinchoninic acid


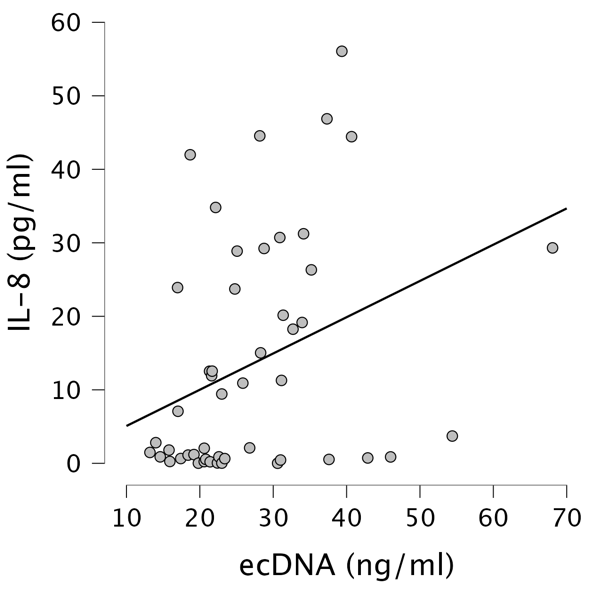


Supplementary figure 1. Correlation of ecDNA levels in blood plasma and IL-8 in patients with MS.


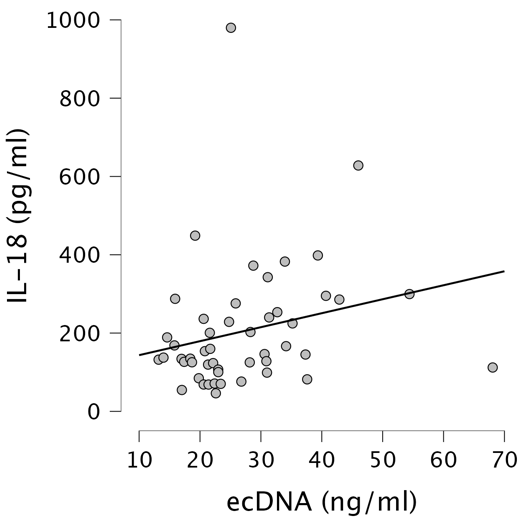


Supplementary figure 2. Correlation of ecDNA levels in blood plasma and IL-18 in patients with MS.


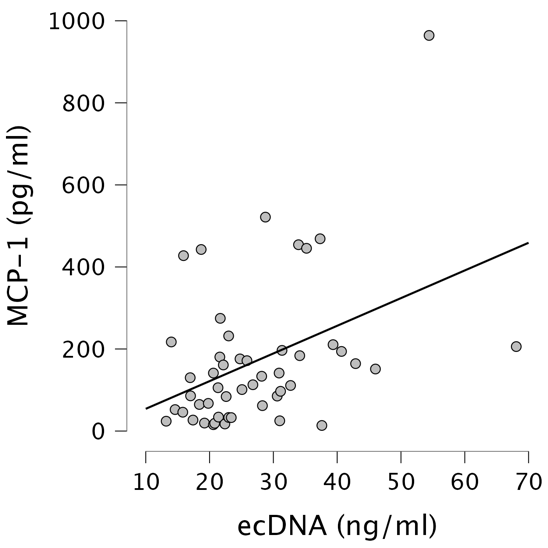


Supplementary figure 3. Correlation of ecDNA levels in blood plasma and MCP-1 in patients with MS.

**
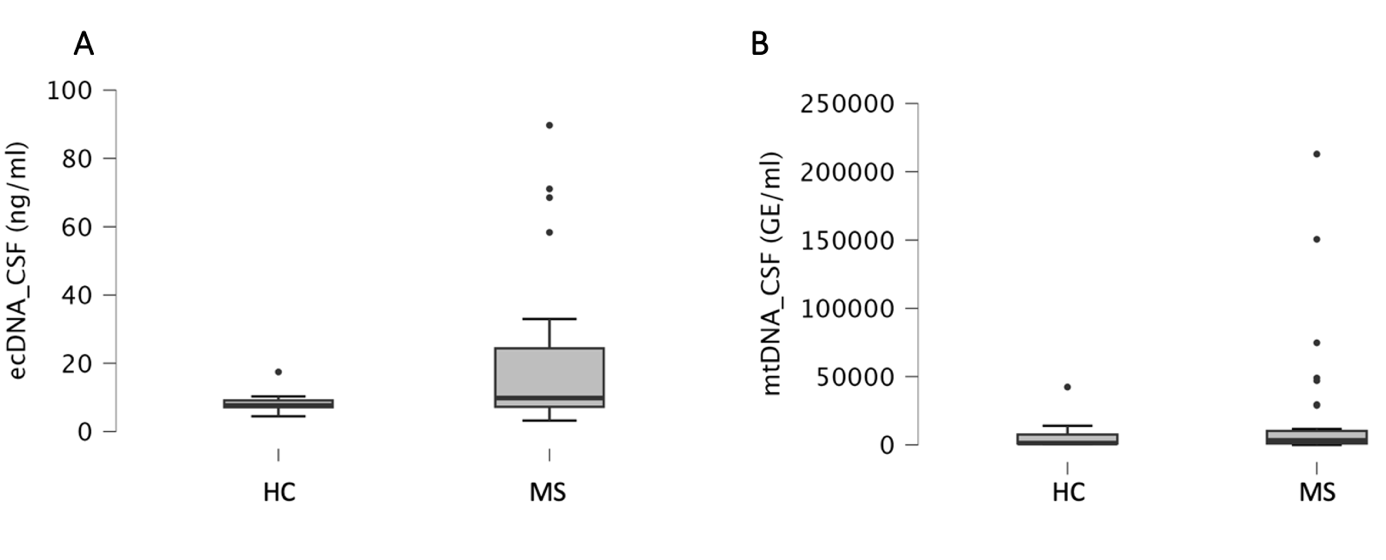
**

Supplementary figure 4. Comparison of CSF ecDNA levels (A) and CSF mtDNA levels (B) between healthy controls (HC) and patients with MS.

**A B**


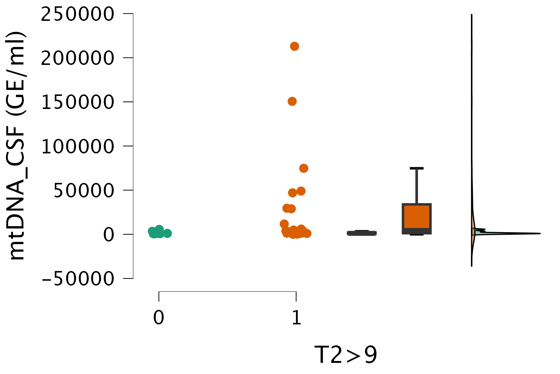

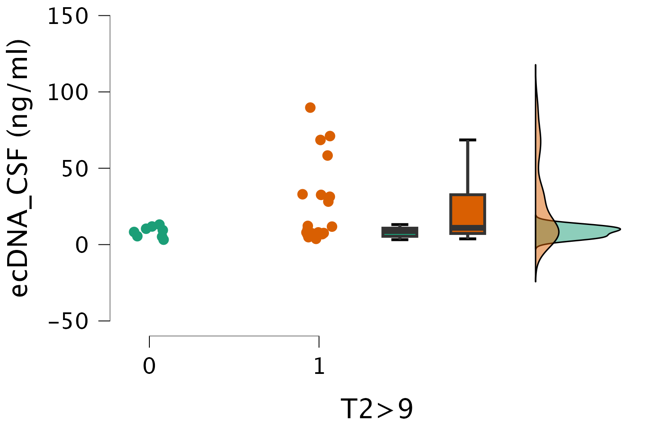


Supplementary figure 5. Comparison of CSF mtDNA levels (A) and CSF ecDNA levels (B) in patients with MS with more than 9 T2 MRI lesion load
